# Supplementary material for: Population Demographics of Owned Dogs in Greater Bangkok and Implications for Free-Roaming Dog Population Management
Source: Animals (Basel). 2025 Apr 29;15(9):1263. doi: 10.3390/ani15091263 (PMC12070910; doi:10.3390/ani15091263)
Supplement: Supplementary file 1 [file animals-15-01263-s001.zip › animals-3526896-supplementary.pdf]

Greater Bangkok KAP survey

Interviewer name

____

Record your current location

____

latitude (x.y °)

____

longitude (x.y °)

____

altitude (m)

____

accuracy (m)

____

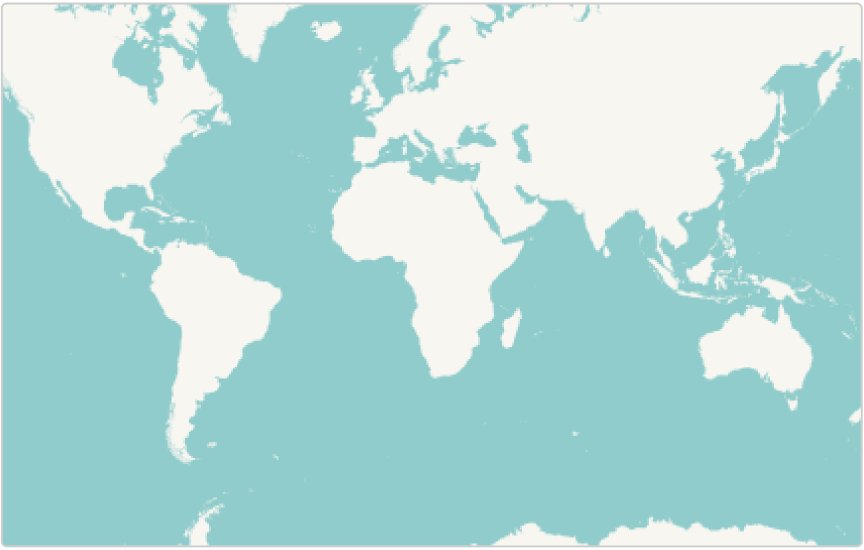

District and sub-district or just district name if in Bangkok

- (selected) Bang Kapi_
- Din Daeng_
- Don Mueang_
- Dusit_
- Huai Khwang_
- Khan Na Yao_
- Khlong San_
- Lak Si_
- Min Buri_
- Pathum Wan_
- Phra Nakhon_
- Prawet_
- Saphan Sung_
- Taling Chan_
- Thawi Watthana_
- Bang Len_Nin Phet
- Bang Len_Bang Len
- Bang Len_Sai Ngam
- Don Tum_Lam Hoei
- Don Tum_Huai Duan
- Kamphaeng Saen_Thung Khwang
- Kamphaeng Saen_Sa Si Mum
- Kamphaeng Saen_Wang Nam Khiao
- Mueang Nakhon Pathom_Thap Luang
- Mueang Nakhon Pathom_Ta Kong
- Mueang Nakhon Pathom_Wang Yen
- Mueang Nakhon Pathom_Bang Khaem
- Nakhon Chai Si_Laem Bua
- Nakhon Chai Si_Lan Tak Fa
- Nakhon Chai Si_Bang Krabao
- Nakhon Chai Si_Nakhon Chai Si
- Nakhon Chai Si_Tha Phraya
- Phutthamonthon_Sala Ya
- Sam Phran_Tha Talat
- Sam Phran_Talat Chinda
- Sam Phran_Bang Chang
- Sam Phran_Rai Khing
- Sam Phran_Tha Kham
- Bang Kruai_Bang Khun Kong
- Bang Kruai_Sala Klang
- Bang Kruai_Bang Khu Wiang
- Bang Yai_Sao Thong Hin
- Mueang Nonthaburi_Bang Phai
- Mueang Nonthaburi_Bang Krang
- Pak Kret_Pak Kret
- Sai Noi_Khlong Khwang
- Sai Noi_Nong Phrao Ngai
- Sai Noi_Sai Yai
- Sai Noi_Khun Si
- Khlong Luang_Khlong Sam
- Khlong Luang_Khlong Si
- Khlong Luang_Khlong Ha
- Khlong Luang_Khlong Chet
- Lam Luk Ka_Lam Luk Ka
- Lam Luk Ka_Khu Khot
- Lat Lum Kaeo_Khlong Phra Udom
- Mueang Pathum Thani_Bang Khayaeng
- Nong Suea_Bueng Ba
- Nong Suea_Sala Khru
- Nong Suea_Bueng Bon
- Nong Suea_Bueng Cham O
- Nong Suea_Bueng Ka Sam
- Nong Suea_Noppharat
- Sam Khok_Chiang Rak Yai
- Sam Khok_Khlong Khwai
- Sam Khok_Sam Khok
- Sam Khok_Thai Ko
- Sam Khok_Krachaeng
- Sam Khok_Ban Pathum
- Sam Khok_Chiang Rak Noi
- Sam Khok_Bang Pho Nuea
- Sam Khok_Bang Toei
- Sam Khok_Ban Ngio
- Sam Khok_Bang Krabue
- Thanyaburi_Rangsit
- Bang Bo_Khlong Dan
- Bang Bo_Preng
- Bang Phli_Bang Pla
- Bang Phli_Bang Kaeo
- Mueang Samut Prakan_Bang Mueang Mai
- Mueang Samut Prakan_Bang Mueang
- Mueang Samut Prakan_Samrong Nuea
- Phra Pradaeng_Bang Ya Phraek
- Phra Pradaeng_Bang Chak
- Phra Pradaeng_Bang Yo
- Phra Pradaeng_Talat
- Ban Phaeo_Kaset Phatthana
- Ban Phaeo_Khlong Tan
- Ban Phaeo_Amphaeng
- Ban Phaeo_Yok Krabat
- Ban Phaeo_Lak Song
- Krathum Baen_Om Noi
- Mueang Samut Sakhon_Phanthai Norasing
- Mueang Samut Sakhon_Na Khok
- Mueang Samut Sakhon_Bang Thorat
- Mueang Samut Sakhon_Bang Nam Chuet
- Mueang Samut Sakhon_Mahachai
- Mueang Samut Sakhon_Tha Sai
- Mueang Samut Sakhon_Tha Chalom
- Mueang Samut Sakhon_Chai Mongkhon

Is there anyone home?

- (●) Yes
- ( ) No

Did they decline to be interviewed immediately?

- ( ) Yes
- (●) No

Read out the consent statement... Do they fulfill the criteria and consent to be interviewed?

Consent - My name is_I am conducting market research so we can understand dogs in your community better. There are no essential questions, you can skip any question they want to. There is no cost to participating and you won't be paid. The data will be shared with organizations that can provide services for dogs and consultants for analysis. You must be at least 18 years old to take part (or if you are younger than 18 years, you can take part if you are supervised by an adult from within your household). You must have lived in this household for at least a year, as we will be asking questions about dogs over the past year. Are you willing to take part in my survey?

- (●) Yes - Adult
- ( ) Yes - Child under 18 years with adult supervision
- ( ) No

Did you have to explain you are from Soi Dog Foundation?

Ideally the interview is completed without them knowing where you are from, but if asked, you must answer honestly

- ( ) Yes
- ( ) No

Part A

What is your age in years?

____

What gender?

Do not ask, just record based on appearance

- ( ) Male
- ( ) Female
- ( ) Unclear

Do you own any dogs?

- (●) Yes
- ( ) No

Did any dogs leave your household within the last year?

- (●) Yes
- ( ) No

How many dogs have left your household within the last year?

____

» Dogs that left

1

What sex was this dog that left?

- ◯ Male
- ◯ Female
- ◯ I don't know

What happened to this dog?

- ◯ Sold
- ◯ Given away
- ◯ Disappeared
- ◯ Died in road accident
- ◯ Died injury
- ◯ Died illness
- ◯ Died rabies
- ◯ Died unknown cause
- ◯ Abandoned temple or street
- ◯ Killed by myself
- ◯ Killed by somoene else
- ◯ Other fate

How old was this dog when it left?

- ◯ Puppy under 6 months
- ◯ Juvenile 6-12 months
- ◯ Adult 1 year and older
- ◯ I don't know

Did you or anyone in your household offer care, such as food, to a street dog that doesn't have an identifiable owner in the last week?

- ◉ Yes
- ◯ No

What kind of care was provided to this street dog or dogs?

- [ ] Food
- [ ] Water
- [ ] Shelter
- [ ] Vaccination
- [ ] Sterilisation
- [ ] Deworming
- [ ] External parasite treatment
- [ ] Affection or friendship
- [ ] Protection from other dogs or people
- [ ] Other

Do you know anyone in your street whose owned dog had puppies in the last 12 months?

- (x) Yes
- ( ) No

What happened to those puppies?

- [ ] They kept them
- [ ] Gave them away as gifts
- [ ] Sold them
- [ ] Left them on the street
- [ ] Left them in a location people use for dumping dogs
- [ ] Gave them to a dog shelter
- [ ] Died of natural causes
- [ ] Killed them
- [ ] Other
- [ ] I don't know

In the last month, have you been annoyed or troubled by a dog or dogs in your neighbourhood?

- (x) Yes
- ( ) No

What was this annoyance or trouble?

- [ ] Noise - barking, howling or crying
- [ ] Pee and poo
- [ ] Make a mess of garbage
- [ ] Bite me or other people
- [ ] Chased me or other people
- [ ] Attacked my pet
- [ ] Attacked my livestock
- [ ] Damaged my crops
- [ ] Sexual behaviour
- [ ] Dog fights
- [ ] Destroy my property
- [ ] Saw a rabid dog
- [ ] Injured or sick dog
- [ ] Concern for puppies
- [ ] Other

Part B

Has anyone in your household been bitten within the last 12 months?

- (●) Yes
- ( ) No

How many bite incidence have there been in the last 12 months?

____

Bite incidence

1

Who was bitten?

- ( ) Male adult (17+ years)
- ( ) Female adult (17+ years)
- ( ) Male child (5-16 years)
- ( ) Female child (5-16 years)
- ( ) Male infant (<5 years)
- ( ) Female infant (<5 years)

Who were they bitten by?

- ◯ Our own dog
- ◯ A dog owned by somone else
- ◯ Community dog
- ◯ A dog we know doesn't belong to anyone and isn't cared for by the community
- ◯ Unknown dog, I don't know whether it was owned or not

What did you do about this bite?

- ◯ Nothing
- ◯ Only treated at home with washing and other home treatments
- ◯ Went to hospital or doctor for medical treatment
- ◯ Other

Part C

How many dogs are free-roaming on your soi today?

ownership irrelevant, question is whether they are roaming unsupervised?

____

What do you think about this number of dogs on your soi?

- ◯ I'm happy with this number of dogs
- ◯ I accept this number of dogs
- ◯ I don't have an opinion on the number of dogs
- ◯ I do not accept this number of dogs
- ◯ I am unhappy with this number of dogs

Over the last 12 months, do you think there has been a change in the number of roaming dogs in your community?

- ◯ Increase
- ◯ No change
- ◯ Decrease

Thinking of the number of dogs on your soi today – and then thinking back to when Prayut Chan-o-cha became Prime Minster in 2014 – which of the following statements is most true?

- ◯ There used to be lots more dogs on my soi
- ◯ There used to be far fewer dogs on my soi
- ◯ The number of dogs is about the same
- ◯ I only moved here recently
- ◯ I don't know

Thinking of your experience with dogs on your soi today – and then thinking back to when Prayut Chan-o-cha became Prime Minster in 2014 – which of the following statements is most true?

- ◯ I used to be more troubled by the dogs on my soi
- ◯ I used to be less troubled by the dogs on my soi
- ◯ The troubles I have with the dogs on my soi are about the same
- ◯ I only moved here recently
- ◯ I don't know

What do you think should be done about roaming dogs?

- ◯ Left alone
- ◯ Sterilisation
- ◯ Taken away to live in a shelter
- ◯ Rehomed as pets
- ◯ Other
- ◯ I don't know

What is this other thing that should be done with roaming dogs?

____

Have you ever visited a dog shelter in Thailand?

- ◯ Yes
- ◯ No

How many dogs do you own?

____

Dog details - repeat for each dog

1

Dog name

____

Age in years

____

Age in months

____

Age in days

____

's age is year(s), month(s) and day(s)

Dog sex

- ○ Male
- ○ Female

Where did you get this dog from?

- ○ Purchased from breeder
- ○ Purchased from market or shop
- ○ Gift
- ○ Adopted from the street
- ○ Adopted from a shelter
- ○ Pup of my own dog
- ○ Other

Did you acquire this dog with the last year?

- ○ Yes
- ○ No, longer than 1 year ago
- ○ I don't know

For how long on a normal day is this dog confined?

Confinement means the dog cannot roam outside the household, it includes confinement to a garden/yard

- ○ Confined all day and all night, never allowed to roam
- ○ Roams at night, confined in day
- ○ Roams in day, confined at night
- ○ Rarely < 2 hrs
- ○ Never confined, dog has freedom to roam outside my property all the time

Was this dog vaccinated against rabies in the last 12 months?

- ○ Yes
- ◉ No
- ○ I don't know

Why is this dog not vaccinated?

Select their main reason

- ◯ Too expensive
- ◯ Too young
- ◯ I don't know where to get my dog vaccinated
- ◯ Vaccination point is too far away
- ◯ I cannot transport my dog to the vaccination point or clinic
- ◯ I cannot handle my dog for vaccination
- ◯ Vaccination is dangerous
- ◯ Not necessary
- ◯ I don't know about dog rabies vaccination
- ◯ Other

What is this other reason not to vaccinate your dog?

Is this dog sterilised?

Sterilisation means permanent, select 'no' if they are temporarily contracepted using hormones

- ◯ Yes
- ◉ No
- ◯ I don't know

Why have you chosen not to sterilise this dog?

Select their main reason

- ◯ Too young
- ◯ Want puppies
- ◯ Want to breed
- ◯ Too expensive
- ◯ No skilled vet / Worried about the safety of the procedure
- ◯ Unnatural / religious objections
- ◯ Unwanted behaviour change
- ◯ Cruel/feel sorry for dog
- ◯ Don't need to as dog confined
- ◯ Don't need to as the dog is male
- ◯ Don't know about sterilisation
- ◯ Use hormonal contraception (injection) instead
- ◯ Other
- ◯ I don't know

What is this other reason?

Where was this dog sterilised?

- ○ Private vet clinic
- ○ Government vet clinic
- ○ NGO vet clinic
- ○ Other

Have you taken this dog to the vet clinic within the last 12 months?

- ◉ Yes
- ○ No
- ○ I don't know

Why did you take your dog to the vet?

- ☐ Health check and preventative treatment - vaccination, deworming or external parasites
- ☐ Sterilisation
- ☐ Disease or illness
- ☐ Injury such as road traffic accident
- ☐ Other

Are you willing to give your name and number for follow-up surveys?

We would like to take your name and number so that we may contact you later with follow-up questions, we would not contact you more than once per year. However, you can also choose to remain anonymous, in which case your name and number will not be recorded.

- ◉ Yes
- ○ No

Name

____

Number

____
